# Supplementary material for: Chronic Systemic SARS-CoV-2 Infection Without Respiratory Involvement in an Immunocompromised Patient
Source: Viruses. 2025 Jan 23;17(2):147. doi: 10.3390/v17020147 (PMC11860550; doi:10.3390/v17020147)
Supplement: Supplementary file 1 [file viruses-17-00147-s001.zip › viruses-3410423-supplementary.pdf]

Table S1. Quality values for the sequences obtained.

Sample	Specimens	Date	Ct	Mean_COV	COV>30X	HQ_SNP	HTZ_SNP	RdRp_Mean_COV	RdRp_COV>30X	Lineage
22061262	Plasma	2022-02-11	38	4640.12	93.19	66	17	5022.93	100	BA.2
22158904	Biopsy	2022-04-26	37	4925.32	99.05	65	16	5202.64	100	BA.2.23
22390729	Nasopharyngeal.	2022-12-19	18	657.86	98.78	91	0	798.10	100	XBB.2.6

COV: Coverage, HQ: High quality, HTZ: Heterozygous.

An in-house bioinformatics pipeline was applied to analyse sequencing data (https://github.com/MG-IiSGM/covid_multianalysis accessed on 06 July 2023). Adapters and low-quality regions were trimmed from paired end reads using fastp (version 0.20.1). Quality control was assessed with fastQC (version v0.11.9). Good-quality reads were mapped to the Wuhan-1 SARS-CoV-2 reference sequence (GenBank accession no. NC_045512.2) with BWA (version 0.7.17-r1188). Ivar (version 1.3.1) was used for variant calling and consensus sequence generation. Lineage annotation was performed with pangolin (version v4.1.2).
